# Cefotaxime-Resistant *Neisseria meningitidis* Sequence Type 4821 Causing Fulminant Meningitis

**DOI:** 10.3201/eid3103.241493

**Published:** 2025-03

**Authors:** Youxing Shao, Mingliang Chen, Jiehao Cai, Yohei Doi, Min Chen, Minggui Wang, Mei Zeng, Qinglan Guo

**Affiliations:** Author affiliations: Institute of Antibiotics, Huashan Hospital, Fudan University, Shanghai, China (Y. Shao, M. Wang, Q. Guo); Key Laboratory of Clinical Pharmacology of Antibiotics, National Heath Commission of the People’s Republic of China, Shanghai (Y. Shao, M. Wang, Q. Guo); Minhang Hospital, Fudan University, Shanghai (M. Chen); Children’s Hospital of Fudan University, Shanghai (J. Cai, M. Zeng); University of Pittsburgh School of Medicine, Pittsburgh, Pennsylvania, USA (Y. Doi); Fujita Health University School of Medicine, Toyoake, Japan (Y. Doi); Shanghai Municipal Center for Disease Control and Prevention, Shanghai (M. Chen)

**Keywords:** *Neisseria meningitidis*, bacteria, meningitis/encephalitis, antimicrobial resistance, sequence type 4821, third-generation cephalosporin resistance, mosaic *penA*, commensal *Neisseria*, horizontal gene transfer, China

## Abstract

We explored the role of commensal *Neisseria* in the emergence of third-generation cephalosporin-resistant *N. meningitidis*. Cefotaxime resistance–conferring *penA795* was prevalent among commensal *Neisseria* isolates in Shanghai, China, and was acquired by a serogroup C quinolone-resistant sequence type 4821 *N. meningitidis,* Nm507, causing fulminant meningitis in an unvaccinated 2-year-old child.

Invasive meningococcal disease (IMD) is a severe infection caused by *Neisseria meningitidis*. Early empiric treatment with penicillin or third-generation cephalosporins (3GCs) including ceftriaxone and cefotaxime, is crucial (*1*). Over the past 2 decades, the spread of the hyperinvasive and quinolone-resistant *N. meningitidis* clone China^CC4821-R1-C/B^ (sublineage L44.1, mainly serogroup C) within clonal complex (CC) 4821 has resulted in ≈70% fluoroquinolone resistance among meningococci in China (*2*). More recently, penicillin nonsusceptiblity has increased rapidly among meningococci in China (*3*–*5*). We recently reported acquisition of penicillin and cefotaxime resistance by CC4821 (*3*).

The *penA* gene (around 1,746 bp, NEIS1753) encodes penicillin binding protein 2 (PBP2), a 2-domain protein (*6*). A 402-bp region (nucleotides 1321–1722, amino acids 441–574) is commonly used to determine *penA* alleles in meningococci. Five well-characterized alterations in the C-terminal catalytic transpeptidase of PBP2 (PBP2-TPase) are primarily responsible for penicillin nonsusceptibility in meningococci (*7*). A series of additional mutations in PBP2-TPase contribute to ceftriaxone resistance in the globally disseminated *N. gonorrhoeae* FC428 clone (*8*,*9*). Three cefotaxime-resistant meningococci isolates were reported in China during 2017–2019 (*3*). The case reported in this article involves, 1 of those 3 isolates, Nm507, serogroup C quinolone-resistant sequence type (ST) 4821 (C: P1.21–2,9:F3–3:ST4821, L44.1), which led to a rare fulminant case of IMD (Appendix). This study was approved by the Institutional Review Board of Children’s Hospital of Fudan University (approval no. 2023–111).

## The Study

A 2-year-old child was initially infected with influenza A virus and subsequently developed purpura fulminans, progressing to septic shock and disseminated intravascular coagulation. Mechanical ventilation, fluid resuscitation, and vasoactive drugs were administered to stabilize his vital signs. After 1 dose of penicillin, antimicrobial therapy was changed to ceftriaxone for 14 days (Appendix Figure 1). The child recovered fully. Previous influenza A virus infection is hypothesized to increase the risk for IMD because it disrupts the nasopharyngeal epithelium and normal flora (*10*).

**Figure 1:**
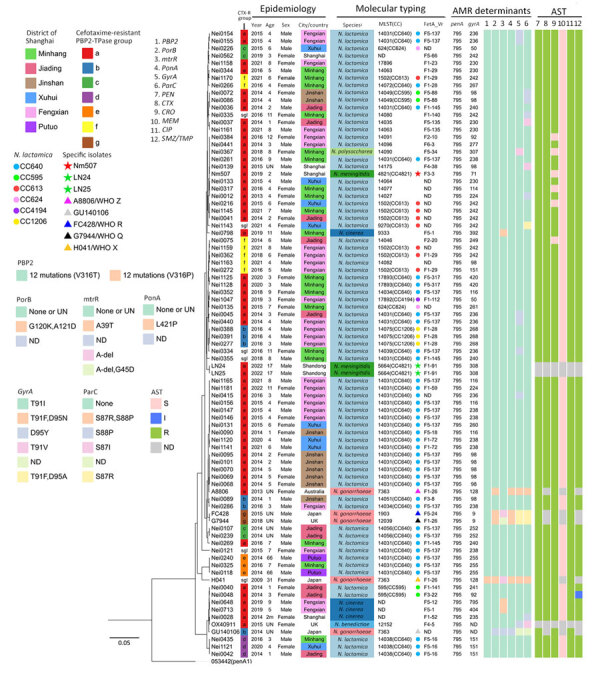
Epidemiologic and molecular characterizations of *penA795*-bearing *Neisseria meningitidis*, *N. gonorrhoeae*, and *Neisseria* commensals in study of cefotaxime-resistant *N. meningitidis* sequence type 4821 causing fulminant meningitis. Epidemiology, molecular typing, antimicrobial resistance determinants, and antimicrobial susceptibility testing results of 85 *penA795*-bearing *Neisseria* isolates are shown. The leftmost tree depicts the phylogeny of the PBP2-TPase region (*penA* 718 to 1,746 bp). Analysis of mutations in antimicrobial resistance-associated genes/determinants is provided in the Appendix. Scale bar indicates number of nucleotide substitutions per site. AMR, antimicrobial resistance; AST, antimicrobial susceptibility testing; CC, clonal complex; CIP, ciprofloxacin; CRO, ceftriaxone; CTX, cefotaxime; MEM, meropenem; MLST, multilocus sequence type; I, intermediate; ND, not determined; PEN, penicillin; R, resistant; sgl, singleton; S, susceptible; UN, unknown.

We isolated 3 *N. meningitidis* isolates from the patient: Nm507 (blood), Nm508 (cerebrospinal fluid), and Nm509 (nasopharynx). Antimicrobial susceptibility testing showed the 3 *N. meningitidis* isolates were resistant to penicillin (MICs 0.75 μg/mL), cefotaxime (MICs 0.25 μg/mL), ciprofloxacin (MICs 0.5 μg/mL), and trimethoprim/sulfamethoxazole (MICs >2/38 μg/mL) and had reduced susceptibility to ceftriaxone (MICs 0.125 μg/mL) and meropenem (MICs 0.047 μg/mL). The 3 isolates were genetically identical and harbored *penA795*, which contained 12 mutations associated with penicillin and 3GCs resistance in the PBP2-TPase. These mutations included 5 classic penicillin-resistance–associated mutations (F504L, A510V, I515V, H541N, and I566V) (*7*) and the A549T mutation, which contributes to penicillin nonsusceptibility (*3*). In addition, they contained 6 mutations found in *N. gonorrhoeae* strain FC428 associated with resistance to 3GCs (A311V, I312M, V316T, T483S, N512Y, and G545S) (*8*,*9*), without any other determinants conferring resistance to 3GCs. Natural transformants, obtained from previous research (*3*), harbored mosaic *penA*, and the segments containing *penA795* were transferred from 3 commensal *Neisseria* species to penicillin- and 3GCs-susceptible recipient *N. meningitidis* Nm040 (penicillin MIC 0.032 µg/mL, cefotaxime MIC 0.008 µg/mL). All natural transformants showed penicillin nonsusceptibility (MIC 0.19–0.38 µg/mL) and cefotaxime resistance (MIC 0.25–0.5 µg/mL). In addition, MICs were elevated by 32–62-fold (0.064–0.125 µg/mL) for ceftriaxone and by 2–5-fold (0.023–0.064 µg/mL) for meropenem compared with recipient Nm040 (ceftriaxone MIC ≤0.002 µg/mL, meropenem MIC 0.012 µg/mL) (Appendix Table 1).

**Figure 2:**
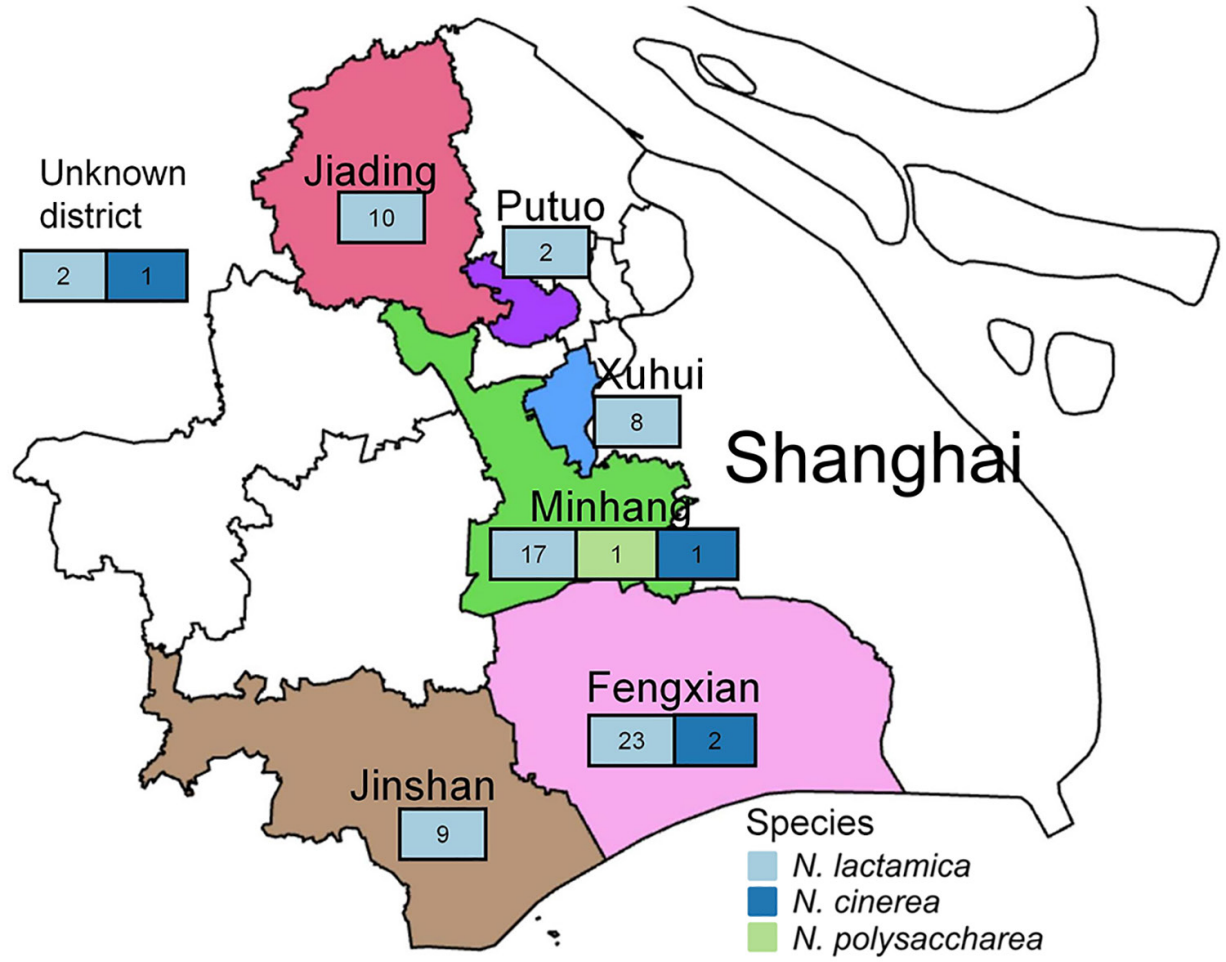
Sampling locations of 76 *penA795*-bearing *Neisseria* commensals in 6 districts of Shanghai, China, in study of cefotaxime-resistant *Neisseria meningitidis* sequence type 4821 causing fulminant meningitis.

**Figure 3:**
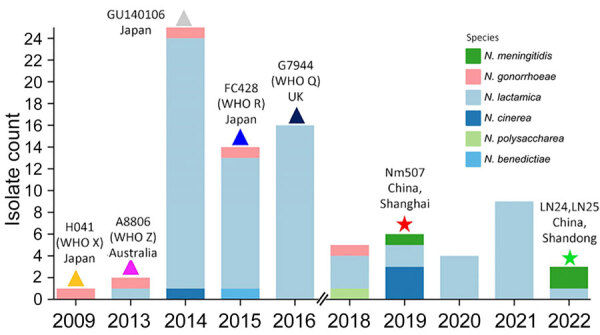
Timeline of 85 *penA795*-bearing *Neisseria* isolates from China and elsewhere during 2009–2022 in study of cefotaxime-resistant *Neisseria meningitidis* sequence type 4821 causing fulminant meningitis. WHO, World Health Organization.

We cataloged the *penA* alleles in 1,032 local commensal *Neisseria* isolates (commensals) and observed *penA795* in 76/1,032 (7.4%) isolates. The 76 *penA795*-bearing commensals included *N. lactamica* (n = 71), *N. cinerea* (n = 4), and *N. polysaccharea* (n = 1) (Figure 1). Among the 71 *N. lactamica* isolates, CC640 dominated (54.9%, 39 isolates; 27 isolates were ST14031). Those *penA*795-bearing commensals were collected from 6 districts of Shanghai (Figures 1, 2); they were isolated from persons of various ages, mainly children 3–6 years of age (n = 42), students 7–11 years of age (n = 18), and children <3 years of age (n = 10), over a 10-year period (2013–2022) (Figures 1, 3). All 76 *penA795*-bearing commensals were resistant to penicillin, cefotaxime, and ciprofloxacin and nonsusceptible (75/76 resistant, 1 intermediate) to trimethoprim/sulfamethoxazole but susceptible to meropenem. Seventy-one isolates (93%, 71/76) were resistant to ceftriaxone (Figure 1; Appendix Table 2).

We identified the *penA795* allele among 6 *Neisseria* species (85 isolates, including 76 from this study) through searches in PubMLST (https://pubmlst.org/neisseria) and PubMed (*8*,*11*–*14*). Of note, *penA795* has been transferred among unrelated *N. gonorrhoeae* clones such as H041 (World Health Organization gonococcal reference strain X [WHO X], 2009, Japan) (*11*), A8806 (WHO Z, 2013, Australia) (*12*), GU140106 (2014, Japan) (*13*), the FC428 international clone (WHO R, 2015, Japan) (*8*), and G7944 (WHO Q, 2018, United Kingdom) (*14*) (Figures 1, 3; Appendix Table 1). Only 3 *penA795*-bearing *N. meningitidis* isolates have been reported: Nm507 from Shanghai in 2019 and LN24 and LN25 (PubMLST identifications 133563 and 133564) from Shandong Province, China, in 2022. LN24 and LN25 exhibit identical molecular characteristics as B: P1.20,23–1: F1–91:ST5664 (CC4821, L44.2) (Figures 1, 3). MIC data for LN24 and LN25 are unavailable. We extracted the full-length *penA* (NEIS1753) sequences from 85 *penA795*-bearing *Neisseria* isolates and divided them at nucleotide position 718, according to the N terminal (*penA* 1–717) and C terminal (*penA* 718–1,746) encompassing the 3 conserved motifs of the PBP2-TPase domain of PBP2 (Appendix Figures 2, 3) (*6*). The +718 to +900 region possessed 1%–28% nucleotide variations. Except for H041, which had an amino acid alternation of V316P, all isolates harbored the 12 resistance-associated mutations in PBP2-TPase described in Nm507 and FC428 (Appendix Table 1, Figure 3). We classified 85 *penA795*-bearing *Neisseria* isolates into 7 groups (a–g) and 6 singletons based on PBP2-TPase sequences associated with cefotaxime resistance (*penA* 901–1,746, covering 12 resistance-associated mutations) (Figure 1). Nm507 shared an identical cefotaxime-resistant PBP2-TPase sequence with 50 *penA795*-bearing commensals, as well as LN24, LN25, and A8806 (CTX-R group a), suggesting likely horizontal gene transfer among different *Neisseria* species, especially from *N. lactamica* to *N. meningitidis*. Natural transformation experiments (*3*) reproduced the horizontal gene transfer event of *N. meningitidis* acquiring the *penA795*-bearing segment from *Neisseria* commensals. 

Commensal *Neisseria*, with a 100% carriage rate, serve as an ideal reservoir of antimicrobial resistance genetic elements for local meningococci. The widespread presence of *penA795*-bearing *N. lactamica* CC640, exhibiting multidrug resistance to penicillin, cefotaxime, ceftriaxone, ciprofloxacin, and trimethoprim-sulfamethoxazole in Shanghai, raises substantial concerns over its role in fostering the emergence of multidrug-resistant *N. meningitidis*. Moreover, the increase of international travel heightens the risk for both meningococcal and multidrug-resistant commensal colonization. Of note, *penA795*-bearing *N. meningitidis* (LN24 and LN25) in Shandong were recovered sharing identical PBP2-TPase sequences associated with cefotaxime resistance with commensals from Shanghai.

## Conclusions

Our study reveals widespread presence of the *penA795* allele, which encodes PBP2-TPase associated with cefotaxime resistance, among *Neisseria* commensals in Shanghai, China. The *penA795* fragment has been captured by the hyperinvasive, quinolone-resistant NmC *N. meningitidis* ST4821, causing life-threatening meningitis. In China, scheduled meningococcal vaccines include the group A polysaccharide vaccine (MPV-A, 2 doses at 6 and 9 months) and the bivalent NmA and NmC polysaccharide vaccine (MPV-AC, administered at 3 and 6 years of age). This patient had received only 2 doses of MPV-A, because MPV-AC is not licensed for young children (<2–3 years of age). A recent study has just reported an outbreak of NmC caused by multidrug-resistant ST4821 isolates in Fiji (*15*). Promoting coverage with meningococcal polysaccharide conjugate vaccine (MPCV), such as MPCV-AC or MPCV-ACYW, will be imperative to effectively reduce illness and deaths caused by *N. meningitidis*, particularly the emerging multidrug-resistant invasive NmC clone.

Appendix Additional information about cefotaxime-resistant *Neisseria meningitidis* ST-4821 causing fulminant meningitis.
