# Supplementary material for: Cefotaxime-Resistant Neisseria meningitidis Sequence Type 4821 Causing Fulminant Meningitis
Source: Emerg Infect Dis. 2025 Mar;31(3):591–5. doi: 10.3201/eid3103.241493 (PMC11878294; doi:10.3201/eid3103.241493)
Supplement: Appendix — Additional information about cefotaxime-resistant Neisseria meningitidis ST-4821 causing fulminant meningitis. [file 24-1493-Techapp-s1.pdf]

Article DOI: https://doi.org/10.3201/eid3103.241493

EID cannot ensure accessibility for supplementary materials supplied by authors. Readers who have difficulty accessing supplementary content should contact the authors for assistance.

Cefotaxime-Resistant *Neisseria meningitidis* ST-4821 Causing Fulminant Meningitis

Appendix

Case Report

In January 2019 (winter), a 2-year-old boy was brought to the emergence room for fever, lethargy, and skin petechiae. The boy had fever (thermal spike 39°C) and cough 5 days earlier. On day four of illness, he was taken to a local hospital for persistent fever and was diagnosed with influenza A virus (IAV) infection (Figure. S1). On day five of his illness, scattered petechiae were noted over his face which then rapidly spread over the whole body while he became increasingly lethargic and irritable.

On arrival, he was very drowsy with body temperature of 38°C, heart rate of 152 beats/per minute, respiratory rate of 28 breaths/per minute, blood pressure of 84/49 mm Hg, cold extremities with the prolonged the capillary refill time of >3s, extensive ecchymosis and purpuric macules distributed across his entire body. The laboratory investigations revealed thrombopenia and significant elevation of markers of inflammation (Figure. S1), coagulation abnormalities, high urea and creatinine levels, hypoxemia (PaO_2, 72.3%) and metabolic acidosis (lactate, 1.9 mmol/L). Chest radiograph revealed bilateral lung infiltrates. The patient was clinically

diagnosed with septic shock, disseminated intravascular coagulation, pneumonia, and renal dysfunction. He received mechanical ventilation, continuous fluid resuscitation, vasoactive drugs, and intravenous penicillin, and was transferred to the intensive care unit immediately. The patient also received oseltamivir, dexamethasone, heparin, and blood products. After 1 dose of intravenous penicillin, the antimicrobial therapy was changed to ceftriaxone (750 mg every 12 hours) and continued for 14 days (Figure. S1). The CSF, blood, and nasal swab cultures all grew *N. meningitidis*. The child was extubated after 4 days on mechanical ventilation. Another febrile episode occurred on day 12 of hospitalization, accompanied with new skin petechiae and ecchymoses. Complete blood cell count showed decrease platelet counts (1~8 × 10^9/L) (Appendix Figure 1). On the clinical ground, he was diagnosed with idiopathic thrombocytopenic purpura following infection. An 8-day course of methylprednisolone was administered and the child fully recovered. He had no recent travel history and had received 2 doses of group A meningococcal polysaccharide vaccines (MPV-A).

Methods

Bacterial Isolates Collection and Typing

N. meningitidis isolates used in this study were collected as part of the routine clinical management of IMD patients in People's Republic of China (*1*). The commensal carriage surveys were conducted by the Shanghai Center for Disease Control and Prevention (CDC), following protocols described in previous studies (*2–5*). Posterior oropharyngeal swabs were collected from over 2200 children (aged <15 years) during 2013 and 2022, including infants (aged <3 years), toddlers in kindergarten (aged 3–6 years), students in primary school (aged 7–11 years), and students in junior high school (aged 12–15 years). In addition, a small number of samples were isolated from senior citizens (aged >65 years). *Neisseria* spp. identification was performed using standard procedures (*2–5*), including Gram staining, oxidase reaction, matrix-assisted laser desorption ionization-time of flight mass spectrometry (MALDI-TOF-MS;

bioMérieux, France), and *rplF* gene sequencing. *N. meningitidis* isolates were sequenced on Illumina HiSeq instrument and submitted to the PubMLST *Neisseria* Database (https://pubmlst.org/neisseria/) (Appendix Table 1). Molecular typing was performed following the nomenclature in the PubMLST database and previous protocols (*2–5*). We used the primers recommended by Taha *et al.* (*6*) to determine the *penA* alleles for *Neisseria* isolates.

Antimicrobial Susceptibility Testing

The MICs (MICs) of penicillin, cefotaxime, ceftriaxone, meropenem, ciprofloxacin, and trimethoprim-sulfamethoxazole were determined using the broth microdilution method. For *N. meningitidis* isolates, we also used the Etest (bioMérieux) to determine MICs. The interpretation of breakpoints followed the 2024 guidelines of the Clinical and Laboratory Standards Institute (CLSI) (*7*). Meningococcal breakpoints were also applied to commensals.

Analysis of Mutations in Antimicrobial Resistance Associated Genes

Five key alterations (F504L, A510V, I515V, H541N, and I566V) and A549T in the PBP2-TPase have been linked to neisserial penicillin resistance (*4*,*6*). Mutations in GyrA (T91 and D95) and ParC (D86 and S87) are associated with quinolone resistance in *N. meningitidis* and *Neisseria lactamica* (*2*,*5*). In *N. gonorrhoeae*, specific alterations in PBP2-TPase (A311V, I312M, V316T/P, T483P/S, A501V/P/T, N512Y, A516G, G542S, G545S, and P551L/S), PorB (G120K and A121N/D alterations), *mtrR* (−35A deletion, +A39T, and G45D), and PonA (L421P alteration) have been implicated in 3GCs resistance (*8*,*9*).

Genetic Transformation

Chromosomal fragments from commensal *Neisseria* isolates were introduced into *N. meningitidis* following a previously established method (*3–5*). Briefly, chromosomal DNA from potential commensal *Neisseria* donor isolates, identified via genomic analysis and carrying the *penA795* allele, was extracted using the Qiagen DNA Minikit (Qiagen, Hilden, Germany) as per the manufacturer's guidelines. The recipient strain Nm040, susceptible to penicillin and 3GCs

(penicillin MIC: 0.032 μg/mL, cefotaxime MIC: 0.008 μg/mL), was grown overnight on Columbia agar (Oxoid, Basingstoke, UK) with 5% sheep blood. This strain was then incubated at 37°C for 5–6 hours with 500 ng of each donor chromosomal DNA in proteose peptone medium (Oxoid) enriched with BBL IsoVitaleX (BD, Sparks, MD, USA). The mixtures were plated on Columbia agar with sheep blood, supplemented with cefotaxime (2-fold dilutions, 0.03–0.5 μg/mL), and incubated at 37°C under 5% CO_2 for 24–48 hours.

REFERENCE

1. Chen M, Guo Q, Wang Y, Zou Y, Wang G, Zhang X, et al. Shifts in the antibiotic susceptibility, serogroups, and clonal complexes of *Neisseria meningitidis* in Shanghai, China: a time trend analysis of the pre-quinolone and quinolone eras. PLoS Med. 2015;12:e1001838, discussion e1001838. PubMed https://doi.org/10.1371/journal.pmed.1001838

2. Shen Y, Chen M. Prevalence, sequence type, and quinolone resistance of *Neisseria lactamica* carried in children younger than 15 years in Shanghai, China. J Infect. 2020;80:61–8. PubMed https://doi.org/10.1016/j.jinf.2019.08.020

3. Shao Y, Chen M, Luo J, Li D, Yuan L, Yang X, et al. Serogroup Y clonal complex 23 meningococcus in China acquiring penicillin resistance from commensal *Neisseria lactamica* species. Antimicrob Agents Chemother. 2022;66:e0238321. PubMed https://doi.org/10.1128/aac.02383-21

4. Chen M, Shao Y, Luo J, Yuan L, Wang M, Chen M, et al. Penicillin and cefotaxime resistance of quinolone-resistant *Neisseria meningitidis* clonal complex 4821, Shanghai, China, 1965–2020. Emerg Infect Dis. 2023;29:341–50. PubMed https://doi.org/10.3201/eid2902.221066

5. Chen M, Zhang C, Zhang X, Chen M. Meningococcal quinolone resistance originated from several commensal *Neisseria* species. Antimicrob Agents Chemother. 2020;64:e01494–19. PubMed https://doi.org/10.1128/AAC.01494-19

6. Taha MK, Vázquez JA, Hong E, Bennett DE, Bertrand S, Bukovski S, et al. Target gene sequencing to characterize the penicillin G susceptibility of *Neisseria meningitidis.* Antimicrob Agents Chemother. 2007;51:2784–92. PubMed https://doi.org/10.1128/AAC.00412-07

7. Clinical and Laboratory Standards Institute. Performance standards for antimicrobial susceptibility testing. 34rd ed. Supplement M102. Wayne, PA: The Institute, 2024.

8. Unemo M, Shafer WM. Antimicrobial resistance in *Neisseria gonorrhoeae* in the 21st century: past, evolution, and future. Clin Microbiol Rev. 2014;27:587–613. PubMed https://doi.org/10.1128/CMR.00010-14

9. Allan-Blitz LT, Adamson PC, Klausner JD. Resistance-guided therapy for *Neisseria gonorrhoeae.* Clin Infect Dis. 2022;75:1655–60. PubMed https://doi.org/10.1093/cid/ciac371

10. Guo Q, Mustapha MM, Chen M, Qu D, Zhang X, Chen M, et al. Evolution of sequence type 4821 clonal complex meningococcal strains in China from prequinolone to quinolone era, 1972–2013. Emerg Infect Dis. 2018;24:683–90. PubMed https://doi.org/10.3201/eid2404.171744

11. Ohnishi M, Golparian D, Shimuta K, Saika T, Hoshina S, Iwasaku K, et al. Is *Neisseria gonorrhoeae* initiating a future era of untreatable gonorrhea?: detailed characterization of the first strain with high-level resistance to ceftriaxone. Antimicrob Agents Chemother. 2011;55:3538–45. PubMed https://doi.org/10.1128/AAC.00325-11

12. Lahra MM, Ryder N, Whiley DM. A new multidrug-resistant strain of *Neisseria gonorrhoeae* in Australia. N Engl J Med. 2014;371:1850–1. PubMed https://doi.org/10.1056/NEJMc1408109

13. Deguchi T, Yasuda M, Hatazaki K, Kameyama K, Horie K, Kato T, et al. New clinical strain of *Neisseria gonorrhoeae* with decreased susceptibility to ceftriaxone, Japan. Emerg Infect Dis. 2016;22:142–4. PubMed https://doi.org/10.3201/eid2201.150868

14. Nakayama S, Shimuta K, Furubayashi K, Kawahata T, Unemo M, Ohnishi M. New ceftriaxone- and multidrug-resistant *Neisseria gonorrhoeae* strain with a novel mosaic penA gene isolated in

Japan. Antimicrob Agents Chemother. 2016;60:4339–41. PubMed https://doi.org/10.1128/AAC.00504-16

15. Eyre DW, Sanderson ND, Lord E, Regisford-Reimmer N, Chau K, Barker L, et al. Gonorrhoea treatment failure caused by a *Neisseria gonorrhoeae* strain with combined ceftriaxone and high-level azithromycin resistance, England, February 2018. Euro Surveill. 2018;23:1800323. PubMed https://doi.org/10.2807/1560-7917.ES.2018.23.27.1800323

16. Tunyasuvunakool K, Adler J, Wu Z, Green T, Zielinski M, Žídek A, et al. Highly accurate protein structure prediction for the human proteome. Nature. 2021;596:590–6. PubMed https://doi.org/10.1038/s41586-021-03828-1

17. Powell AJ, Tomberg J, Deacon AM, Nicholas RA, Davies C. Crystal structures of penicillin-binding protein 2 from penicillin-susceptible and -resistant strains of *Neisseria gonorrhoeae* reveal an unexpectedly subtle mechanism for antibiotic resistance. J Biol Chem. 2009;284:1202–12. PubMed https://doi.org/10.1074/jbc.M805761200

Appendix Table 1. Characteristics and Antimicrobial Susceptibility Testing Results for the *penA795*-bearing *Neisseria* Isolates in Natural Transformation Experiments, *N. meningitidis* and *N. gonorrhoeae* Clinical isolates.

Isolate	Species	Characteristics	MIC (µg/mL) and susceptibility * PEN	CTX	CRO	MEM	*penA* allele (NG-STAR *penA* allele) †	NEIS1753 allele (PBP2-TPase mutations motif)‡	Other 3GCs determinants §	Reference	PubMLST ID/GenBank accession number
Nm040	*N. meningitidis*	Transformation recipient	0.032 (S)	0.008 (S)	≤0.002 (S)	0.012 (S)	*penA1*	NEIS1753_267 (wild-type PBP2)	none	(*10*)	58130
Nm507	*N. meningitidis*	Clinical ST-4821 isolate from blood	0.75 (R)	0.25 (R)	0.125 (62-fold)	0.047 (4-fold)	*penA795*	NEIS1753_3923 (5Pen^{NS}-*V311-M312-T316-S483-Y512-S545-T549)*	none	(*4*), this study	72262
Nm508	*N. meningitidis*	Clinical ST-4821 isolate from CSF	0.75 (R)	0.25 (R)	0.125 (62-fold)	0.047 (4-fold)					106173
Nm509	*N. meningitidis*	Clinical ST-4821 isolate from nasal swab	0.75 (R)	0.25 (R)	0.125 (62-fold)	0.047 (4-fold)					106174
Nei012	*N. lactamica*	Transformation donor 1	1.5 (R)	1 (R)	0.25 (R)	0.047 (4-fold)	*penA795*	NEIS1753_3350 (5Pen^{NS}-*V311-M312-T316-S483-Y512-S545-T549)*	none	(*4*), this study	84195
Nm040Nei012T1–6	*N. meningitidis*	Transformant of Nm040 with Nei012	0.25 (I)	0.5 (R)	0.094 (48-fold)	0.047 (4-fold)	*penA795*	5Pen^{NS}-*V311-M312-T316-S483-Y512-S545-T549*			111273
Nm040Nei012T1n-2	*N. meningitidis*		0.19 (I)	0.5 (R)	0.094 (48-fold)	0.047 (4-fold)					111274
Nm040Nei012T1n-3	*N. meningitidis*		0.25 (I)	0.5 (R)	0.094 (48-fold)	0.047 (4-fold)					111275
Nei028	*N. cinerea*	Transformation donor 2	1 (R)	1 (R)	0.75 (R)	0.064 (5-fold)	*penA795*	NEIS1753_2976 (5Pen^{NS}-*V311-M312-T316-S483-Y512-S545-T549)*	none	(*4*), this study	84201
Nm040Nei028T1n-1	*N. meningitidis*	Transformant of Nm040 with Nei028	0.38 (I)	0.5 (R)	0.125 (62-fold)	0.064 (5-fold)	*penA795*	5Pen^{NS}-*V311-M312-T316-S483-Y512-S545-T549*			111276
Nm040Nei028T1n-2	*N. meningitidis*		0.25 (I)	0.5 (R)	0.125 (62-fold)	0.064 (5-fold)					111277

Isolate	Species	Characteristics	MIC (μg/mL) and susceptibility * PEN	CTX	CRO	MEM	penA allele (NG-STAR penA allele) †	NEIS1753 allele (PBP2-TPase mutations motif)‡	Other 3GCs determinants §	Reference	PubMLST ID/GenBank accession number
Nm040Nei028T1n-3	*N. meningitidis*		0.25 (I)	0.5 (R)	0.094 (48-fold)	0.047 (4-fold)					111278
Nei367	*N. polysacchera*	Transformation donor 3	0.75 (R)	0.5 (R)	0.19 (95-fold)	0.094 (5-fold)	*penA795*	NEIS1753_4539 (5Pen^{NS}-*V311-M312-T316-S483-Y512-S545-T549)*	none	(*4*), this study	84258
Nm040Nei367T1–1	*N. meningitidis*	Transformant of Nm040 with Nei367	0.25 (I)	0.25 (R)	0.094 (48-fold)	0.032 (3-fold)	*penA795*	5Pen^{NS}-*V311-M312-T316-S483-Y512-S545-T549*			111288
Nm040Nei367T1–2	*N. meningitidis*		0.38 (I)	0.25 (R)	0.125 (62-fold)	0.047 (4-fold)					111289
Nm040Nei367T1–3	*N. meningitidis*		0.19(I)	0.25 (R)	0.064 (32-fold)	0.047 (4-fold)					111290
Nei798 ¶	*N. cinerea*	Transformation donor 4	0.25 (I)	0.25 (R)	0.094 (48-fold)	0.047 (4-fold)	*penA795*	NEIS1753_4685 (5Pen^{NS}-*V311-M312-T316-S483-Y512-S545-T549)*	none	(*4*), this study	105711
Nm040Nei798T1–1	*N. meningitidis*	Transformant of Nm040 with Nei798	0.19 (I)	0.25 (R)	0.064 (32-fold)	0.047 (4-fold)	*penA795*	5Pen^{NS}-*V311-M312-T316-S483-Y512-S545-T549*			111291
Nm040Nei798T1–3	*N. meningitidis*		0.25 (I)	0.25 (R)	0.094 (48-fold)	0.023 (2-fold)					111292
Nm040Nei798T1–4	*N. meningitidis*		0.25 (I)	0.25 (R)	0.125 (62-fold)	0.047 (4-fold)					111293
H041	*N. gonorrhoeae*	The first documented ceftriaxone-resistant gonococcus, Japan, 2009, WHO X **	4 (R)	2 (R)	2 (R)	0.125	*penA795* (*penA-37.001*)	NEIS1753_545‖ (5Pen^{NS}-*V311-M312-P316-S483-Y512-S545-T549)*	L421P, G120K, and A121D, −35 A del	(*11*)	88865
A8806	*N. gonorrhoeae*	The third ceftriaxone-resistant gonococcus,	1 (R)	ND	0.5 (R)	ND	*penA795* (*penA-64.001*)	NEIS1753_1551	L421P, G120K, and A121D	(*12*)	61379

Isolate	Species	Characteristics	MIC (µg/mL) and susceptibility * PEN	CTX	CRO	MEM	*penA* allele (NG-STAR *penA* allele) †	NEIS1753 allele (PBP2-TPase mutations motif)‡	Other 3GCs determinants §	Reference	PubMLST ID/GenBank accession number
		Australia, 2013, WHO Z**						(5Pen^{NS}-*V311-M312-T316-S483-Y512-S545-T549)*			
GU140106	*N. gonorrhoeae*	Japan, 2014	2 (R)	ND	0.5 (R)	ND	*penA795* (*penA-59.001*)	NEIS1753_1547 (5Pen^{NS}-*V311-M312-T316-S483-Y512-S545-T549)*	ND*††*	(*13*)	LC056026 (*penA* sequence)
FC428	*N. gonorrhoeae*	International ceftriaxone-resistant FC428 clone, Japan, 2015, WHO R**	>32 (R)	ND	0.5 (R)	ND	*penA795* (*penA-60.001*)	NEIS1753_1548 (5Pen^{NS}-*V311-M312-T316-S483-Y512-S545-T549)*	L421P, G120K, and A121D, −35 A del	(*14*)	108261
G7944	*N. gonorrhoeae*	Ceftriaxone-resistant and high-level azithromycin-resistant gonococcus, UK, 2018, WHO Q**	ND	ND	0.5 (R)	ND	*penA795* (*penA-60.001*)	NEIS1753_1548 (5Pen^{NS}-*V311-M312-T316-S483-Y512-S545-T549)*	L421P, G120K and A121D, −35 A del and G45D	(*15*)	157714 (GCA_900411645.1)

*The interpretation of breakpoints was according to the 2024 guidelines of the CLSI methods (*7*): penicillin, MIC ≤0.06 µg/mL as susceptible, MIC 0.12–0.25 µg/mL as intermediate, and MIC ≥0.5 µg/mL as resistant; cefotaxime and ceftriaxone MIC ≤0.125 µg/mL as susceptible; meropenem MIC ≤0.25 µg/mL as susceptible. The magnitude of MIC increase (ceftriaxone and meropenem) was compared to the transformation recipient strain Nm040. CRO, ceftriaxone; CTX, cefotaxime; I, intermediate; MEM, meropenem; ND, not detected; PEN, penicillin; R, resistant; S, susceptible; WHO, World Health Organization.

†Meningococcal *penA* typing scheme (402-bp *penA* allele, nucleotides 1321–1722) according PubMLST *Neisseria* Database (https://pubmlst.org/neisseria/). NG-STAR, *N. gonorrhoeae* Sequence Typing for Antimicrobial Resistance molecular typing scheme (https://ngstar.canada.ca).

‡A concatenated sequence or motif was used to outline the combinations of mutations in PBP2-TPase. For example, 5Pen^{NS}-V311-M312-T316-S483-Y512-S545-T549 motif represents the twelve penicillin- and 3GCs-resistance-associated amino acids mutations in PBP2-TPase: F504L, A510V, I515V, H541N, and I566V (5Pen^{NS}), A311V, I312M, V316T, T483S, N512Y, G545S, and A549T

§Other 3GCs-resistance-associated determinants: PonA, L421P alteration; PorB, G120K and A120D alterations; *mtrR*, deletion of upstream nt 35A (−35 A del), insertion of A39T (+A39T) and G45D.

¶Nei798 was reclassified as *N. cinerea* by ribosomal MLST (In our previous study it was mistaken as *N. subflava*).

‖H041, with amino acid alternation of V316P.

**WHO *N. gonorrhoeae* reference strains.

††Whole-genome sequencing data unavailable.

Appendix Table 2. Antimicrobial susceptibility testing results for 76 *penA795*-bearing commensal *Neisseria* isolates*

MIC (μg/mL)†	PEN	CTX	CRO	MEM	MIC (μg/mL)†	CIP	MIC (μg/mL)†	SMZ-TMP
<0.06	0	0	0	16	0.125	15	0.25/4.75	1
0.06	0	0	0	50	0.25	36	0.5/9.5	7
0.125	0	0	5	9	0.5	22	1/19	15
0.25	0	2	54	1	>0.5	3	2/38	38
0.5	0	15	16	0			>2/38	15
1	19	40	1	0				
2	49	13	0	0				
>2	8	6	0	0				
MIC_{50} (μg/mL)‡	2	1	0.25	0.06	MIC_{50} (μg/mL)‡	0.25	MIC_{50} (μg/mL)‡	2/38
MIC_{90} (μg/mL)‡	>2	2	0.5	0.125	MIC_{90} (μg/mL)‡	0.5	MIC_{90} (μg/mL)‡	>2/38
Susceptibility	76 (R)	76 (R)	71 (R), 5 (S)	76 (S)	Susceptibility	76 (R)	Susceptibility	1(I), 75 (R)
Resistance rate	100%	100%	71/76, 93.4%	0	Resistance rate	100%	Non-susceptibility rate	100%

*CIP, ciprofloxacin; CTX, cefotaxime; CRO, ceftriaxone; MEM, meropenem; PEN, penicillin; SMZ-TMP, trimethoprim/sulfamethoxazole.

†MIC, MIC. The interpretation of breakpoints according to the 2024 guidelines of the CLSI methods (*7*): penicillin, MIC ≤0.06 μg/mL as susceptible (S), MIC 0.12–0.25 μg/mL as intermediate (I), and MIC ≥0.5 μg/mL as resistant (R); cefotaxime and cefatriaxone MIC ≤0.125 μg/mL as susceptible; meropenem MIC ≤0.25 μg/mL as susceptible; ciprofloxacin MIC ≤0.03 μg/mL as susceptible, MIC 0.06 μg/mL as intermediate, and MIC ≥0.125 μg/mL as resistant; trimethoprim/sulfamethoxazole MIC ≤0.12/2.4 μg/mL as susceptible, MIC 0.25/4.75 μg/mL as intermediate, and MIC ≥0.5/9.5 μg/mL as resistant.

‡MIC_{50} and MIC_{90}, MICs at which 50% and 90% of the tested isolates are inhibited respectively.

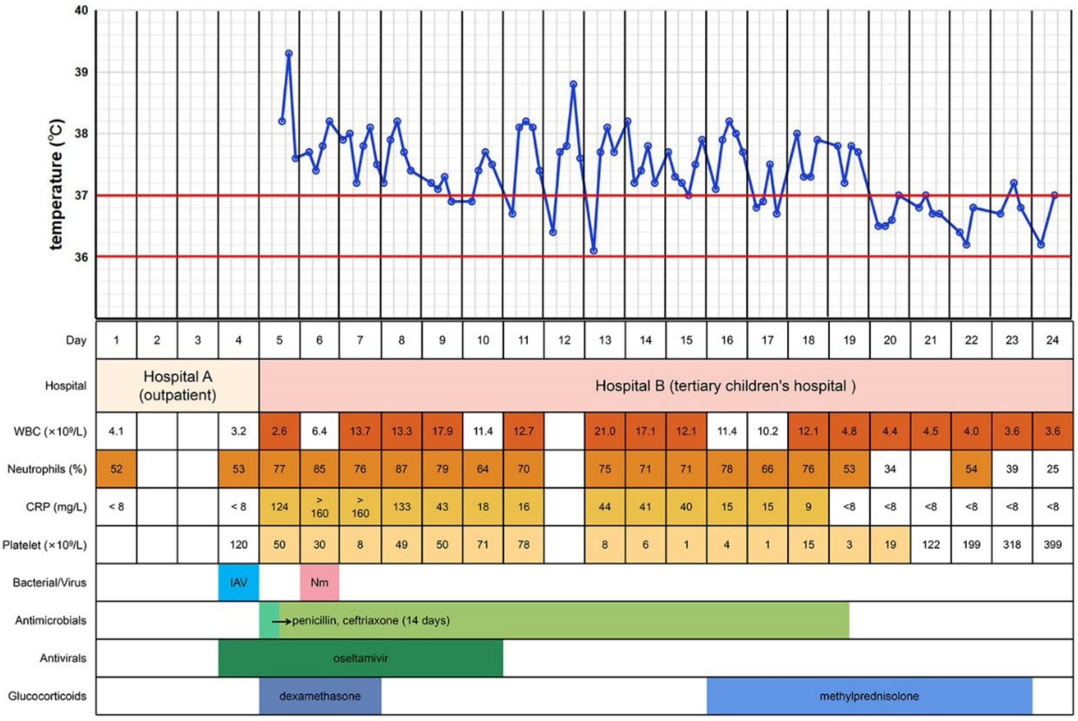

Day	1	2	3	4	5	6	7	8	9	10	11	12	13	14	15	16	17	18	19	20	21	22	23	24
Hospital	Hospital A (outpatient)				Hospital B (tertiary children's hospital)																			
WBC (×10⁹/L)	4.1			3.2	2.6	6.4	13.7	13.3	17.9	11.4	12.7		21.0	17.1	12.1	11.4	10.2	12.1	4.8	4.4	4.5	4.0	3.6	3.6
Neutrophils (%)	52			53	77	85	76	87	79	64	70		75	71	71	78	66	76	53	34		54	39	25
CRP (mg/L)	< 8			< 8	124	> 160	> 160	133	43	18	16		44	41	40	15	15	9	<8	<8	<8	<8	<8	<8
Platelet (×10⁹/L)				120	50	30	8	49	50	71	78		8	6	1	4	1	15	3	19	122	199	318	399
Bacterial/Virus				IAV		Nm																		
Antimicrobials					→penicillin, ceftriaxone (14 days)																			
Antivirals				oseltamivir																				
Glucocorticoids					dexamethasone											methylprednisolone								

Appendix Figure 1. Clinical Information of Fulminant IMD Case. Temperature curve, key laboratory findings and pharmacological interventions are indicated according to day of illness and hospitalization. Abnormal laboratory findings are highlighted. leukocyte, leukocyte; CRP, C-reactive protein; IAV, influenza A virus; Nm, *Neisseria meningitidis*.

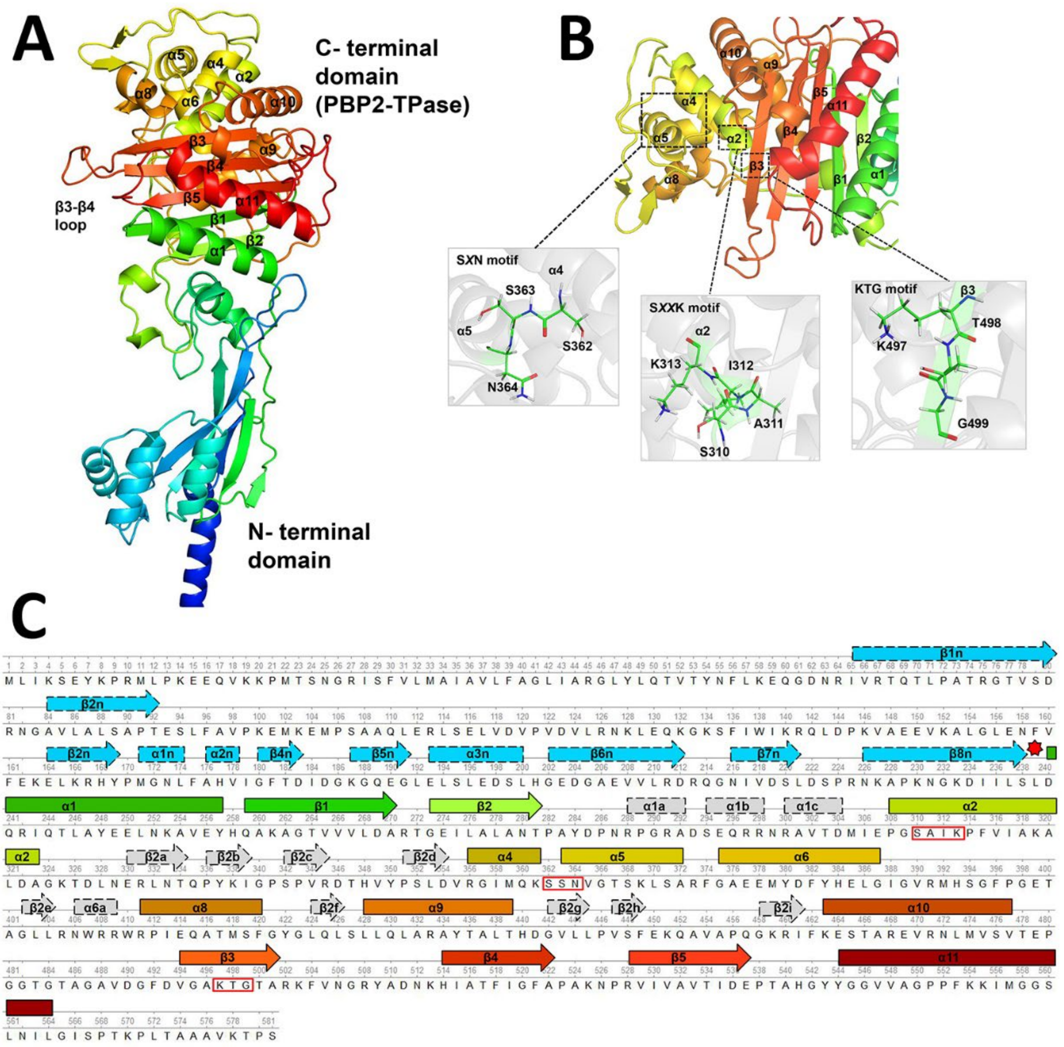

Appendix Figure 2. The structure of *N. meningitidis* PBP2. (A) A ribbon representation of wild-type (WT) PBP2 from *N. meningitidis* isolate Nm040, predicted using AlphaFold 2 (https://alphafold.ebi.ac.uk) (*16*). The structure is color-ramped from the N terminal to the C-terminal direction. Secondary structure elements are labeled according to Powell *et al*. (*17*), as described in *N. gonorrhoeae*. (B) The C-terminal/PBP2-TPase domain of Nm040 contains three conserved motifs: S*XX*K (Ser-310, Lys-313), S*X*N (Ser-362, Asn-364), and KTG (Lys-497, Thr-498, and Gly-499), where "*X*" represents a variable amino acid. (C) The amino acid sequence of wild-type PBP2 from *N. meningitidis* isolate Nm040. The division of the N terminal and C-terminal domains at nucleotide position 718 is indicated by a red heptagram symbol. N terminal and C-terminal regions that could not be well-modeled are represented by dashed lines, colored in blue and gray, respectively. The conserved motifs are highlighted by red boxes.

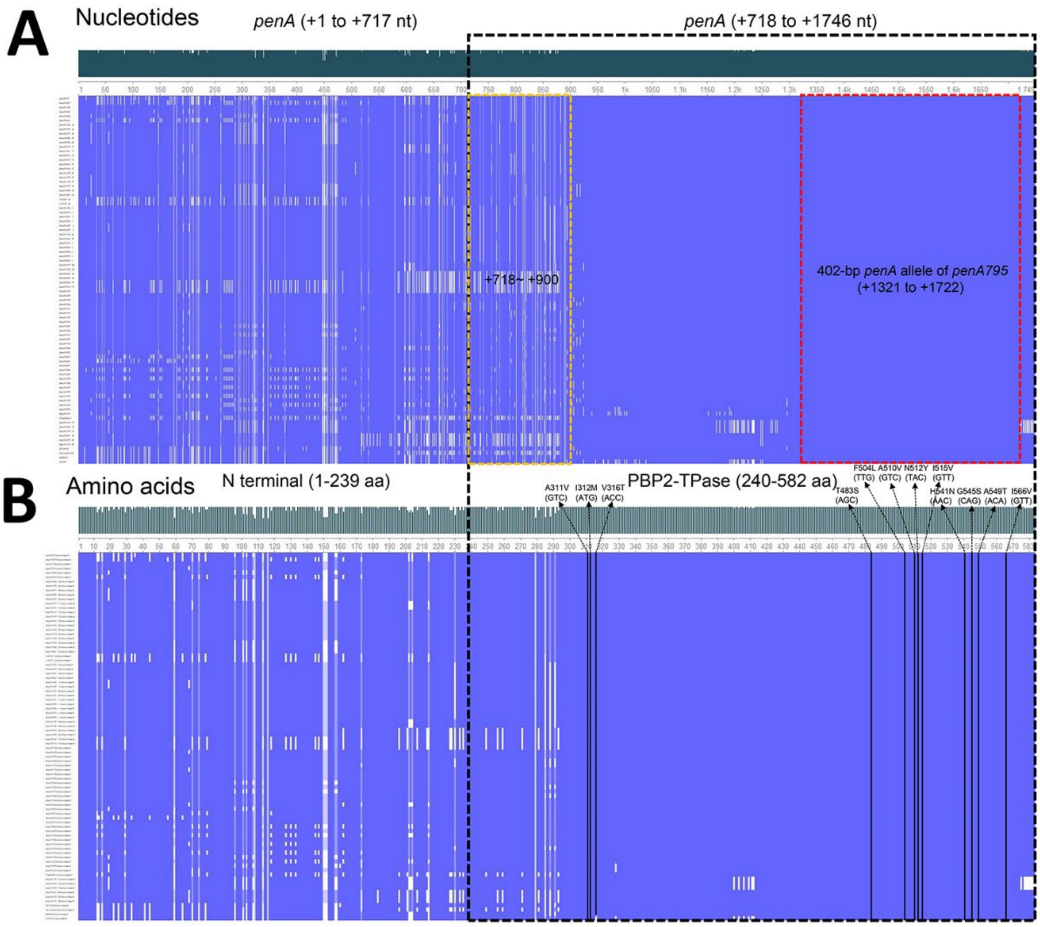

Appendix Figure 3. Nucleotides and amino acids sequences alignment of 85 *penA795*-bearing *Neisseria* isolates including *N. meningitidis* isolates (Nm507, LN24, and LN24), commensal *Neisseria* isolates, and *N. gonorrhoeae* isolates (FC428/G7944, GU140106, A8806, and H041). The deep purple representing complete (100%) nucleotide or amino acid sequence identity and regions with less than 50% identity shown in white. NEIS1753 was divided at nucleotide position +718, with the +1 to +717 region (amino acids 1–239) designated as the N terminal and the +718 to +1746 region (amino acids 240–582) as the PBP2-TPase domain (outlined in a black dotted box). The +718 to +900 region possessed 1%–28% nucleotide variations, is outlined in a yellow dotted box. The 402-bp *penA795* allele is boxed in red. The 12 resistance-associated amino acid mutations (F504L, A510V, I515V, H541N, I566V, A549T,, A311V, I312M, V316T, T483S, N512Y, and G545S) in PBP2-TPase are indicated by black lines.
